# Prevalence of anemia and associated factors among pregnant women in Adigrat General Hospital, Tigrai, northern Ethiopia, 2018

**DOI:** 10.1186/s13104-019-4347-4

**Published:** 2019-05-31

**Authors:** Brhane Berhe, Fitsum Mardu, Haftom Legese, Aderajew Gebrewahd, Guesh Gebremariam, Kebede Tesfay, Getachew Kahsu, Hadush Negash, Gebre Adhanom

**Affiliations:** Department of Medical Laboratory Science, College of Medicine and Health Science, Adigrat University, Adigrat, Ethiopia

**Keywords:** Anemia, Ethiopia, Factors, Pregnant women

## Abstract

**Objectives:**

Anemia remains a major public health problem in Ethiopia, which causes maternal and fetal severe consequences. In Tigrai, there are limited literatures on prevalence of anemia and associated factors among pregnant women. Thus, a hospital based cross-sectional study was conducted to determine the prevalence and associated factors of anemia in Adigrat General Hospital. Data was analyzed and computed using SPSS version 22. *p* value = 0.05 at 95% confidence interval was considered statistically significant.

**Results:**

Overall prevalence of Anemia among the pregnant women attending Adigrat General Hospital was 7.9%. About 62.5% and 37.5% of the anemic women were with mild (Hgb: 10.0–10.9 g/d1) and moderate (Hgb: 7–9.9 g/dl) type respectively. Factors like, residing in rural areas increases risk of anemia by 6 times (AOR = 6, 95% CI 1.34, 27.6, p = 0.019), participants having current blood loss (AOR = 3.4, 95% CI 1.16, 10.2, p = 0.026), having history of recent abortion (AOR = 7.9, 95% CI 2.23, 28.1, p = 0.001) and gestational age in the third trimester (AOR = 4.9, 95% CI 1.39, 17.6, p = 0.013) were statistically associated with anemia. Generally, prevalence of anemia is found to be low in the study area. However, it should be given due attention. Therefore, strong endeavor is needed to control anemia among pregnant women by assessing different micronutrient deficiencies for further prevention.

## Introduction

Anemia is a condition defined with less hemoglobin (Hgb) level than the normal range in the body, which decreases oxygen-carrying capacity of red blood cells to tissues [1]. World Health Organization (WHO) and Center of Disease Control and Prevention (CDC) definitions for anemia differ with age, sex and pregnancy status. The classification is as follows: children 6 months to 5 years anemia is defined as a Hgb level < 11 g/dl, children 5–11 years Hgb < 11.5 g/dl, adult males Hgb < 13 g/dl; non-pregnant women Hgb < 12 g/dl and pregnant women Hgb < 11 g/dl [2, 3].

Globally, Anemia is one of the public health concerns, which affects 32.4 million (38.2%) pregnant women around the world. Particularly, common in South East Asia (48.7%) [4, 5]. Worldwide, it has been reported that nearly 510,000 maternal deaths occur per year associated with childbirth or early post-partum. Approximately 20% of maternal death is caused by anemia; with majority of deaths occurred in developing countries [6].

Anemia is the main cause of morbidity and mortality among pregnant women in developing countries with maternal and fetal consequences [7], which leads to premature births [8], low birth weight [9], fetal cognitive impairment, and death [10, 11].

According to the WHO report of 2008, in Africa, 57.1% of the pregnant women were anemic [12]. Moreover, anemia among pregnant women is having a severe public health problem in Ethiopia with an overall prevalence of 62.7%. Seventeen percent of Ethiopian women in the reproductive age group are anemic and 22% of them women were pregnant [13–16].

The reason of anemia during pregnancy in developing countries includes nutritional deficiencies of iron, folate, and vitamin B12 and parasitic diseases, such as malaria and hookworm. The relative contribution of each of these factors to anemia varies greatly by geographical location, season, and dietary practices [7]. Despite the efforts made by the government and other stakeholders, anemia during pregnancy is still a public health problem in Ethiopia. Since there is limitation of information in the study area, this study was aimed to determine the prevalence and associated factors of anemia in pregnant women in Adigrat General Hospital.

## Main text

### Study design, period and area

Hospital based cross-sectional study was conducted in Adigrat General Hospital from April-September 2018. The Hospital is found in Adigrat town, located around 905 km north of Addis Ababa (capital city of Ethiopia) at an latitude and longitude of 14°16′N 39°27′E, with an elevation of 2457 m (8061 ft) above sea level. Based on the 2007 Census conducted by the Central Statistical Agency of Ethiopia (CSA), the town has a total population of 57,588, people (26,010 are men and 31,578 women). Currently, the Hospital is serving as a teaching hospital, emergency, inpatient and outpatients services for more than 600,000 people who live in eastern zones of Tigrai and Afar regional state. The Hospital has high prevalence of intestinal protozoan parasitic infection.

### Study participants

The study participants were pregnant women greater than 18 years of age and given informed consent who were requested for blood and stool examination in the Hospital during the study period. Convenience sampling technique was employed to enroll the study participants.

### Sample size

A total of 304 participants were determined using single population proportion formula by assuming: 95% level of confidence, 5% margin of error and P (proportion) of 0.235 [3] and non-response rate of 10%.

The degree for severity of anemia in pregnancy was classified into three as per WHO criteria [12]:

- Mild anemia: 10.0–10.9 g/dl
- Moderate anemia: 7.0–9.9 g/dl
- Severe anemia: < 7.0 g/dl

#### Method of data collection

##### Questionnaire

Data on sociodemographic characteristics of study participants and determinant factors of anemia were collected using interviewer based questionnaire by taught data collectors.

##### Stool examination

Labeled stool cup with leak proof covers possessing respective sequential numbers were offered for the study participants. Faecal specimens were carried out by wet mount.

##### Wet mount technique

Fresh stool samples (about 2 mg of stool) were placed on a slide with wooden applicator, emulsified with a drop of physiological saline (0.85%) enclosed with cover slide and examined at 10× and 40× microscopic objectives [17].

##### Blood examination

Labeled venous or heparinized blood samples giving sequential numbers of the study participants were used. Blood samples were used for Hemoglobin determination (by using HemoCue). Thin smear was prepared to observe morphology of the red blood cells.

##### Hemoglobin determination

A venous blood sample was taken, filled to micro cuvette, Wipe off excess blood from the outside of the micro cuvette tip, and then placed in the cuvette holder of the device for measuring hemoglobin concentration [17].

##### Red blood cell morphology

Blood sample was taken from the study participants, a drop of blood was placed on new slide, and thin smear was prepared. After being air-dried, labeled with identification number, the smear of the slide was fixed with absolute methanol and the smear was stained with giemsa solution based on the standard operational procedures (SOPs) and examined at 10× and 100× microscopic objectives [17].

### Quality control

Questionnaires were pre-tested prior to the actual data collection. The collected data were checked for consistency and completeness daily. All the laboratory procedures were conducted as per the standard operating procedures (SOPs).

### Statistical analysis

Data were entered and analyzed using SPSS version 22. Then, summarized using descriptive statistics. Bi-variate and multi-variate regression tests were employed to measure the strength of association between dependent and independent variables. Variables with p < 0.20 in the bi-variate logistic regression were transferred to multi-variate regression analysis to compute AOR. p-value less than 0.05 was considered statistical significant.

#### Ethical consideration

The study was approved by college of health Sciences Research Ethical review committee of Adigrat University, Ethiopia (consent ref. Number AGU/CMHS/096/2018APROVAL dated 18/02/2018. Official letter was obtained from Tigrai Regional Health Bureau to Adigrat General Hospital. Permission was also obtained from Administrator of Adigrat general Hospital. Furthermore, after explaining the importance of study, an informed written consent was obtained from study participants. No name was mentioned during the entire data collection and identification was based on the unique identification number given for each questionnaire and corresponding specimen. Confidentiality of information (results) was kept between the study participant, data collector/investigator and authorized physician. Those study participants who were anemic and showed intestinal parasites were reported to physician to treat according to the national protocol.

### Results

#### Socio-demographic characteristics

A total of 304 pregnant women were included in the study. The mean age of the study participants was 25.3 ± 5.1 (ranged from 18 to 41 years). Out of 304 participants, 217 (71.4%) were living in urban areas and the rest 87 (28.6%) were rural dwellers. More than Half, 235 (77.3%), of the study participants were housewife followed by self-employee, 39 (12.8%) and governmental employee, 30 (9.9%) (Table 1).

**Table 1:** Socio-demographic characteristics of participants and outcome of different factors on the prevalence of anemia in pregnant women attending Adigrat General Hospital, northern Ethiopia, from April to September 2018

Variables	Frequency N (%)	Anemia prevalence	COR (95% CI)	p-value	AOR (95% CI)	p-value
		Positive (%)				
Age (in years)						
18–25	169 (55.6)	5.32	1			
26–34	117 (38.5)	10.26	0.40 (0.2, 1.2)	0.100		
> 34	18 (5.9)	16.67	0.28 (0.06, 1.1)	0.070		
Educational level						
Illiterate	32 (13.8)	43.75	0.87 (0.7, 2.3)	0.700		
1–8	87 (28.6)	3.45	0.50 (0.9, 3.5)	0.350		
9–12	160 (52.6)	2.50	0.40 (0.061, 5.03)	0.900		
> 12	15 (4.9)	–	1			
Residence						
Urban	213 (70.1)	3.75	1		1	
Rural	91 (29.9)	17.58	8.70 (2.01, 38.4)	0.004	6.00 (1.34, 27.6)	0.019
Occupation						
Housewife	235 (77.3)	7.65	0.39 (0.051, 3.04)	0.370		
Self employed	39 (12.8)	7.69	1.30 (0.08, 21.8)	0.850		
Governmental	30 (9.9)	10.00	1			
Pregnancy gap (last and current pregnancy)						
1 year	37 (12.2)	32.43	0.02 (0.005, 0.08)	0.000	0.19 (0.02, 1.70)	0.138
2 years	42 (13.8)	7.14	1.13 (0.12, 11.3)	0.900		
3 years	42 (13.8)	7.14	0.50 (0.09, 3.5)	0.500		
> 3 years	72 (23.7)	4.17	1.90 (0.19, 18)	0.600		
First pregnancy	111 (36.5)	2.70	1		1	
Iron supplements						
Yes	190 (62.5)	–	1		1	
No	114 (37.5)	7.90	0.08 (0.02, 0.31)	0.000	0.06 (0.01, 1.22)	0.089
Presence of current blood loss						
Yes	43 (14.1)	41.86	5.90 (2.17, 16.2)	0.001	3.40 (1.16, 10.2)	0.026
No	261 (85.9)	2.29	1		1	
Trimester (weeks)						
1	60 (19.7)	–	1		1	
2	96 (31.6)	3.12	2.00 (0.79, 12)	0.005	0.14 (0.65, 9.12)	0.070
3	148 (48.7)	14.18	5.00 (1.49, 17.67)	0.010	4.90 (1.39, 17.6)	0.013
Recent history of abortion						
Yes	88 (29)	17.04	9.60 (2.76, 33.2)	0.000	7.90 (2.23, 28.1)	0.001
No	216 (71)	4.17	1		1	
Current history of malaria						
Yes	42 (13.8)	14.28	0.04 (0.01, 0.1)	0.000	0.08 (0.02, 1.26)	0.061
No	262 (86.2)	6.87	1			
History of coffee (the last 3 months)						
Yes	212 (69.7)	8.01	0.12 (0.014, 0.81)	0.030	0.09 (0.010, 1.84)	0.054
No	92 (30.3)	7.61	1			
Home delivery (previous pregnancy delivery)						
Yes	43 (14.1)	6.97	0.05 (0.02, 0.13)	0.000	0.26 (0.04, 1.60)	0.145
No	261 (85.9)	8.01	1			

COR, crude odds ratio, CI, confidence interval, AOR, adjusted odds ratio); 1 (referent); − (no)

#### Laboratory findings

**Fig. 1:**
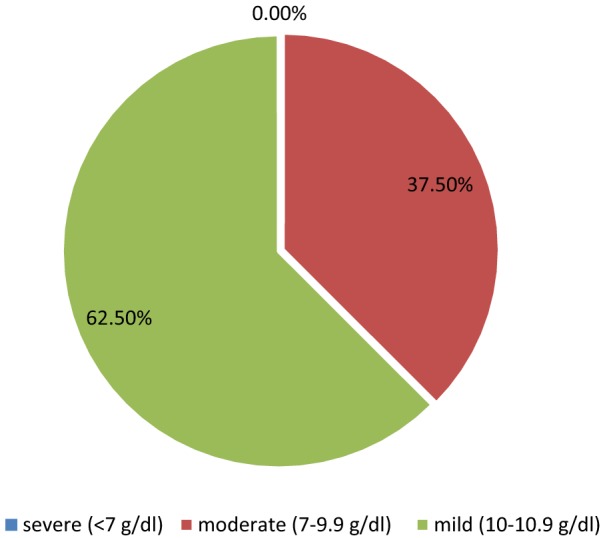
Illustrates the prevalence of the degree of anemia among pregnant women attending Adigrat General Hospital, North Ethiopia, April–September 2018

Among 304 study participants, 24 (7.9%) were anemic (Hgb: < 11 g/dl). The general distribution of anemia was 15 (62.5%) microcytic hypochromic, 6 (25.0%) normocytic hypochromic and 3 (12.5%) macrocytic hypochromic based on the morphology of red blood cells. *Entamoeba histolytica* 24 (7.9%), *Giardia lamblia* and both *Entamoeba histolytica/dispar* and *Giardia lamblia* 15 (4.9%) were among intestinal parasites detected from stool of the pregnant women (Table 2). The majority of anemic cases 62.5% (15/24) showed mild type of anemia (Fig. 1).

**Table 2:** Laboratory finding of the pregnant women attending Adigrat General Hospital, northern Ethiopia, April–September 2018

Hematocrit value (Hgb value)	Number of cases (%)
Anemic < 33% (< 11 gm/dl)	24 (7.9)
Not-anemic > 33% (> 11 gm/dl)	280 (92.1)
Red blood examination	
Microcytic–hypochromic	15 (62.5)
Normocytic–hypocromic	6 (25)
Macrocytic–hypocromic	3 (12.5)
Stool examination	
Entamoeba histolytica/dispar	24 (7.9)
Giardia lamblia	15 (4.9)
Entamoeba histolytica/dispar and Giardia lamblia	15 (4.9)
No ova or parasite	250 (82.2)

#### Factors associated with anemia

All variables with p value < 0.2 (20%) in the bivariate analysis were enter and analyzed by multivariate logistic regression. Study participants those who were residing in rural areas (AOR = 6.0, 95 CI 1.34, 27.6, p = 0.019), women participants responded having current blood loss (AOR = 3.4, 95 CI 1.16, 10.2, p = 0.026), history of recent abortion (AOR = 7.9, 95 CI 2.23, 28.1, p = 0.001) and women in third trimester gestational age (AOR = 4.9, 95 CI 1.39, 17.6, p = 0.013) were factors found statistical significant association with anemia. Though, not statistically significant, prevalence of anemia was higher among those who didn’t use iron supplement, women who gave birth after a year, among illiterate and housewife (Table 1).

### Discussion

The prevalence of anemia in the present study was 7.9%. This was in line with previous studies conducted in Debre Berhan, 9.7% [18], Sudan, 10% [19], Addis Ababa, 11.6% [20] and Iran, 13.6% [21]. However, our finding was observed to be lower than other similar studies [1, 22–27] and in South-East Ethiopia, 27.9% [28]. The difference might be due to geographical variation, differences in socioeconomic status, dietary habits of the study participants [20] and pregnant women with iron supplementation were not excluded in this study. Additionally, the lower occurrence of anemia in this study might be attributed by decreased prevalence of hemoparasites like Malaria in the study area.

In this study, the majority of anemic cases, 62.5% (15/24) were mild type followed by 37.5% (9/24) moderate cases of anemia. A similar provision was reported in Kenya (62.5% and 37.5%) [24] and Nepal (67.1%, and 28.6%) [29] in which majority of the cases was mild anemia followed by moderate anemia respectively. In contrast to this report, study performed in Kenya (70.7% and 26.3%) [30] and southern Ethiopia (60% and 34.3%) [31] majority of the anemia case was moderate followed by mild correspondingly.

History of blood loss (AOR = 3.4, 95 CI 1.16, 10.2, p = 0.026) was significantly associated with the occurrence of anemia. This study was consistent with studies conducted in different parts Ethiopia [31–33]. Similar to a report from South western Ethiopia, history of abortion (AOR = 7.9, 95 CI 2.23, 28.1, p = 0.001) was significantly associated with anemia [33]. This might be due to increased loss of blood which depletes stored iron that leads extra requirement of iron than the usual [34].

Moreover, elevated anemia was found with rural residence and third trimester of gestational age. The pregnant women of rural residence were about 6 times high likely to be anemic than the urban dwellers. The risk of getting anemia was also 4.9 times higher among women in the third trimester of gestational age. This study was parallel with the report in Pakistan [35], India [36], Libya [37], Nepal [38], eastern Ethiopia [39] and northwest Ethiopia [40]. In contrast to this study; as reported in western Nepal [29] and Nigeria [41], the distribution of anemia was higher in second trimester of gestational age.

### Conclusions

This study revealed that the prevalence of anemia among pregnant women was relatively low compared to the findings of other reports in Ethiopia. Rural residence, history of abortion, current blood loss and third trimester gestational age were statistical significant associated factors with anemia in this study. Therefore, further large scale longitudinal studies should be done in respect to the importance of regular visit to maternal care centers and health education promotion programs regarding the cause and prevention of anemia among pregnant women by assessing micronutrients and other causal related factors for anemia.

### Limitation of the study

The study was institutional based study. Further study should be conducted based on community level to make this finding stronger. Besides, the study was restricted cross-sectional study design and didn’t address every nutritional variable.

## Data Availability

The data sets used and/or analyzed during the current study are available from the corresponding author on reasonable request.

## Abbreviations

ANC: antenatal care
CDC: Center of Disease Control and Prevention
CSA: Central Statistical Agency
Hgb: hemoglobin
SPSS: statistical product and service solution
SOPs: standard operating procedures
WHO: World Health Organization
